# Exploring Traditional and Complementary Medicine Approaches for Fractured Bones: A Systematic Review

**DOI:** 10.31661/gmj.v13i.3227

**Published:** 2024-03-01

**Authors:** Naghmeh Yazdi, Mehdi Salehi, Fereshteh Ghorat, Mohammad Hashem Hashempur

**Affiliations:** ^1^ Department of Persian Medicine and Pharmacy, Faculty of Pharmacy, Ahvaz Jundishapur University of Medical Sciences, Ahvaz, Iran; ^2^ Traditional and Complementary Medicine Research Center (TCMRC), Department of Traditional Medicine, School of Medicine, Arak University of Medical Sciences, Arak, Iran; ^3^ Non-Communicable Diseases Research Center, Sabzevar University of Medical Sciences, Sabzevar, Iran; ^4^ Research Center for Traditional Medicine and History of Medicine, Department of Persian Medicine, School of Medicine, Shiraz University of Medical Sciences, Shiraz, Iran

**Keywords:** Bone Fracture, Traditional Medicine, Complementary Medicine, Traditional Persian Medicine, Systematic Review

## Abstract

Background: The objective of this review is to provide a comprehensive summary of published clinical studies that have examined the effects of different traditional and complementary medicine (TACM) interventions on patients with various types of bone fractures. Materials and Methods: A systematic search was conducted in major databases including Web of Science, PubMed, Cochrane library, and Scopus. The search encompassed studies published from the inception of these databases until October 20, 2023. The inclusion criteria encompassed original research papers that evaluated the outcomes of patients with any type of bone fracture who received TACM interventions. Results: Out of the initial 952 search results, a total of six papers met the eligibility criteria and were included in this review. Among these, four studies focused on biologically-based TACM interventions, primarily herbal formulations. The remaining two studies examined energy-based TACM, specifically auricular acupressure and electromagnetic intervention. Conclusion: The findings of this review suggest that the studied TACM modalities demonstrate promising efficacy and safety for patients with fractured bone. However, it is important to note that most of the included studies had limitations in terms of small sample sizes and short follow-up durations.

## Introduction

Bone fractures are a prevalent global health concern, contributing to significant disability, morbidity, and mortality, which places a substantial burden on healthcare systems [1][2]. Despite their high incidence, there remains a scarcity of comprehensive studies on the epidemiology of bone fractures. Existing research suggests an estimated worldwide incidence ranging from 9.0 to 22.8 per 1000 population per year [3]. Notably, bones possess remarkable regenerative capabilities in response to injury, making them unique among tissues. The reparative process of bone fractures involves distinct stages, including inflammation, soft and hard callus formation, and remodeling [4][5]. The choice of treatment for fractures is dependent on several factors, including the type and location of the fracture, as well as individual patient characteristics. Commonly used conventional treatments include reduction and traction, immobilization using various casting techniques, and, in certain cases, open reduction and internal fixation [6][7].

Fracture healing is influenced by innate and adaptive immune functions, as well as the stability of the fixation [8][9][10][11]. Several approaches have been investigated to promote bone healing, including bone marrow grafting, fibroblast growth factor-2, platelet-derived growth factors, Wnt family proteins, and parathyroid hormone [9]. Additionally, extensive research has been conducted on the role of various nutrients such as calcium, zinc, and vitamins D, A, and C in the healing of bone fractures [12].

In recent years, there has been an increasing focus in the scientific literature on traditional and complementary medicine (TACM) interventions to enhance the healing process in patients with bone fractures [13][14]. Furthermore, studies have documented the utilization of TACM by individuals with fractures. For example, a study by Liao et al. (2015) revealed that around 5 percent of individuals with recent bone fractures used traditional Chinese medicine as a complementary treatment [15]. Moreover, a hospital-based study in Taiwan examined the use of Chinese herbal products during various stages of fracture recovery, indicating that patients received an average of three compound herbal medicines and six single medicinal herbs [16]. Another study by Sprague et al. (2007) in Canada reported that 35 percent of bone fracture patients employed TACM [17].

Although there is existing literature on TACM interventions and their utilization by individuals with bone fractures, there is currently no comprehensive overview of previously conducted interventional studies in this domain. Therefore, the objective of this review is to present a synopsis of any clinical studies that have been published and have assessed the impacts of different TACM treatments on patients with various types of bone fractures.

## Materials and Methods

Literature Search

A systematic search was conducted in Web of Science, PubMed, Cochrane library and Scopus databases to identify eligible articles published from the inception of the databases until October 20th, 2023. The following keywords were used for this review: bone fracture, complementary and alternative medicine, integrative medicine, complementary therapies, folk medicine, herbal medicine, medicinal herbs, herbal product, herbal therapy, herbal remedies, phytotherapy, medicinal plants, herbal supplements, manual therapy, traditional therapy, Persian medicine, and traditional medicine.

Inclusion and Exclusion Criteria

Two independent researchers reviewed the bibliographies (MHH and NY) of all retrieved papers. Additionally, the reference lists of included studies and relevant secondary research, such as review studies, were carefully examined to identify any potentially missed results from the systematic searches. Only original research reports that allocated patients with any type of bone fracture to TACM interventions were included. Articles published in the English language were considered for data extraction. Duplicated papers were removed from the study. Any disagreements between the researchers were resolved through group discussion.

Data Extraction

The full text of eligible papers was thoroughly reviewed for data extraction. The following information was recorded: first author’s name, publication date, location of fractures, number of patients assigned to each study arm, and description of the prescribed interventions for each arm (including frequency, duration, dose for pharmaceutical interventions, and timing of interventions). Other information reported in our systematic review included outcome measures (primary and secondary outcomes), main results of the interventions (including P-values, if reported), and any adverse events reported in the TACM intervention group. It is important to note that any missing information from the aforementioned categories was reported as "not mentioned" in the findings.

Quality Assessment

We used the Cochrane’s ROBINS-I tool to evaluate the risk of bias in the studies. This tool allowed for the assessment of seven domains related to study implementation and methodology, including random sequence generation, allocation concealment, blinding of participants and researchers, blinding of outcome assessor, incomplete outcome data, selective reporting, and other bias.

## Results

**Figure-1:**
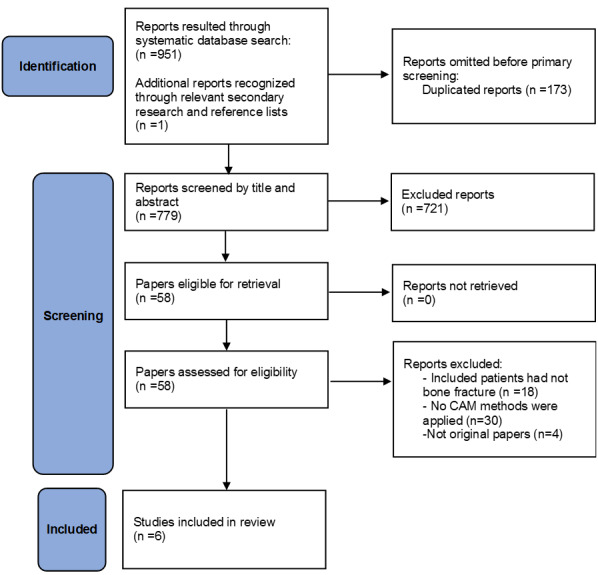
Figure 1. PRISMA flowchart illustrating the selection process of the review.

**Table:** Table1. Main characteristics of the included studies

**Study**	**Population**	**Interventions**	**Outcome assessment**	**Findings**	**TACMs’ adverse events**
**Sadeghi et al. (2020), Iran [18] **	Tibial shaft fracture, 80 patients in each group	Two 500 mg capsules of *momiai* OR placebo (an hour before meal) for 4 weeks after surgery	X-ray radiographies each 4 weeks	The mean days of bone union were 129 and 153 days in the *momiai* and placebo group, respectively (P<0.049).	Mild/moderate headache in two patients in the *momiai* group
**Ram et al. (2013), India [19] **	Jaw (mandible and/or middle one-third) fracture, 50 patients in each group	Antibiotic therapy (5-7 days) alone or in combination with *Septilin * tablet 3 times/day and *Geriforte* tablet 2 times/day (7 days)	Physical examination and radiographies until the complete healing, adverse events, and complications	Clinical union in the intervention group was 1 week (average) faster than the control group. In addition, post-surgical complications (e.g. swelling and tenderness at the site of the fracture, implant failure, and oronasal communication) were not detected in the intervention group.	Not mentioned.
**Sharrard (1990), England [22] **	Tibial shaft fracture with the delayed union, 20 and 25 patients in the intervention and control groups, respectively	Immobilization with plaster, in combination with active electromagnetic stimulation units OR dummy control units for 12 weeks	Radiologic and orthopedic evaluation for bone union	No progress to union in 10 and 23 patients (P=0.002) in the intervention and control group, respectively (based on radiologic findings) and 11 and 22 patients (P=0.02) in the intervention and control group, respectively (based on an orthopedic evaluation)	Not mentioned.
**Nakae** **et al. (2012), Japan [20] **	Rib fracture, 85 patients in each group	*Jidabokuippo* OR nonsteroidal anti-inflammatory drugs (NSAIDs) until the pain reduced to less than half of the initial pain intensity	Treatment duration (as days) and healthcare expenditure (as US $)	The treatment duration was 7 and 14 days in the *jidabokuippo* and NSAIDs groups, respectively (P=0.0003). The healthcare expenditure was 6.29 vs. 19.54 US $ in the jidabokuippo and NSAIDs groups, respectively (P<0.0001).	Unacceptable taste reported by 1 patient
**Nakae** **et al. (2015), Japan [21] **	Extremities fracture, 50 patients	Five to 7.5 g of *jidabokuippo* granules (2-3 times/day, depending on the patients’ weight) within 3 days of fracture	Treatment efficiency (any other medication was not needed)	Eighty-eight percent of patients did not require any other medications. Twelve patients changed their medication or added other medications for symptom alleviation.	No adverse reactions were reported.
**Barker et al. (2006), Austria [23] **	Hip fracture, 18 and 20 patients in the intervention and sham groups, respectively	Pre-hospital admission bilateral auricular acupressure (with 1-mm acupressure plastic beads) at acupressure OR sham points, respectively	Pain and anxiety scores, heart rate, and blood pressure change	Patients in the true intervention group had lower pain (P=0.0001) and anxiety (P=0.018) scores, and heart rate(P=0.0001) on arrival at the hospital, compared to the sham group. There was no significant difference regarding systolic and diastolic blood pressure.	Not mentioned

Included Studies

After applying the inclusion and exclusion criteria, a total of 6 papers remained from the initial 952 search results. Figure-1 presents the PRISMA flowchart of the study, illustrating the number of papers at each stage of the selection process.

Data Synthesis

Table-1 provides a summary of the main characteristics of each of the included studies. Four out of six studies focused on biologically-based TACM interventions [18][19][20][21], including the use of mummy (momiai in Persian) as mineral intervention, Septilin and Geriforte as herbal ayurvedic formulations, and two studies on jidabokuippo as a traditional Japanese medicine. The remaining two studies focused on energy-based TACM interventions, including auricular acupressure and electromagnetic stimulation [22][23].

Quality Assessment

**Figure-2:**
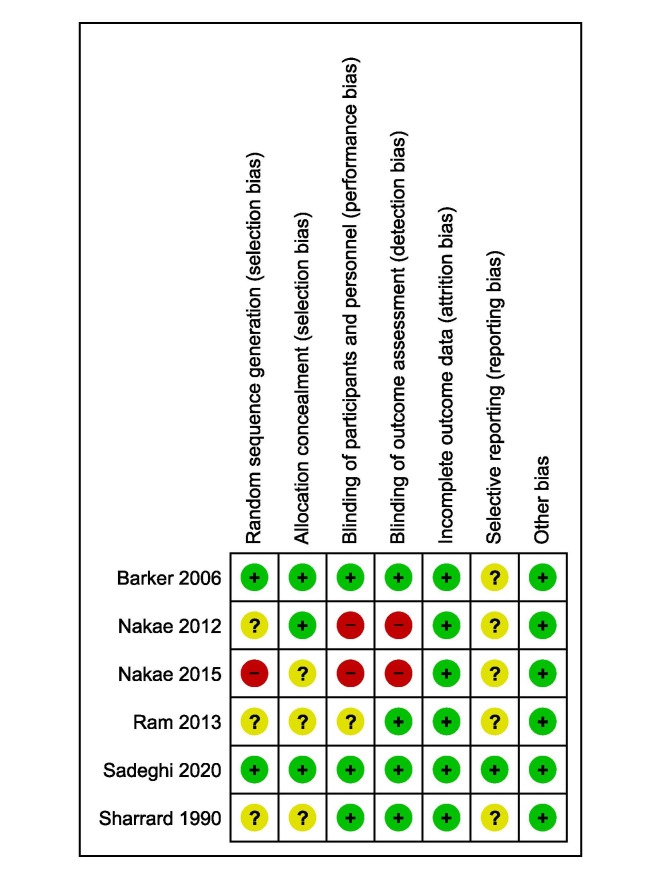
Figure 2. The summary of risk of bias assessment

In five studies, certain methodological domains did not meet the Cochrane criteria for risk of bias, resulting in a high risk of bias rating (Figure-2). Only one study adhered to all of Cochrane’s standards for high-quality research design and was rated as good quality [18]. Furthermore, Barker and colleagues’ study was of medium quality, with low risk of bias in all domains except for an unclear risk of bias related to reporting bias. The most common issue observed was selective reporting, and insufficient detail was provided on random sequence generation in most of the studies.

## Discussion

This systematic review provides a comprehensive overview of TACM interventions utilized for patients with bone fractures, focusing on their impact on pain management, acceleration of bone union, and post-surgery complications. The review identified a significant proportion of studies (four out of six) that investigated biologically-based TACM interventions, including mineral-based treatments such as mummy or momiai, herbal ayurvedic formulations like Septilin and Geriforte, and traditional Japanese medicine jidabokuippo. Additionally, two studies examined energy-based TACM interventions, including auricular acupressure and electromagnetic stimulation, highlighting the diverse range of TACM modalities employed in the context of bone fracture healing. The quality assessment of the included studies revealed that the majority of the studies had a high risk of bias, with only one study meeting all of Cochrane’s criteria for high-quality research design. The findings of this assessment suggest that there is a need for more rigorous study designs and transparent reporting to improve the quality of evidence on TACM interventions for bone fractures.

The most common issue observed in the studies was selective reporting, indicating that the studies may have selectively reported outcomes based on their statistical significance, which can lead to overestimation of treatment effects. Additionally, insufficient detail was provided on random sequence generation in most of the studies, which can increase the risk of selection bias.

The limitations of the studies included in this systematic review highlight the need for more robust and transparent reporting of TACM interventions for bone fractures. Future studies should adhere to high-quality research design standards, including randomization, blinding, and transparent reporting of outcomes. Moreover, studies should report on potential sources of bias to enable the assessment of the quality of the evidence. Nowadays, TACM treatments are welcomed by different health systems worldwide. Moreover, patients have a significant interest in them for a wide variety of acute complaints, chronic diseases and well-being purposes [24][25][26][27][28][29][30][31]. Therefore, it is necessary to make some policies for setting an increasing trend in evidence-based TACM [32][33][34][35][36][37][38].

There are so many instances of the benefits of making TACMs evidence-based and integrating them into health systems. For instance, traditional bonesetters in Africa have great popularity among inhabitants [39].

There are different complications among more than half of individuals with a bone fracture who are treated by these traditional healers [40][41].

According to some reports, a substantial decline was found in the rate of complications (e.g. gangrenous limbs, non-union fractures, and soft tissue infections) when traditional bonesetters were trained and hosted by the governmental health systems [42][43]. Similarly, traditional Persian manuscripts offer a wealth of knowledge on natural products for the healing of bone fractures, yet there is a notable gap between traditional knowledge and evidence-based understanding of these remedies. While Persian resources provide extensive data, scientific examinations of these traditional options are limited [44]. Bridging this gap through clinical trials and modern experimental designs is essential to gain a better understanding of the efficacy and safety of traditional Persian remedies for bone fracture healing, ultimately enhancing the evidence base for their utilization. In light of the increasing interest in TACM interventions and their potential benefits, it is imperative to address the gaps in evidence and policy to ensure the safe and effective integration of traditional and complementary approaches into mainstream healthcare systems. This systematic review highlights the need for further research, including well-designed clinical trials, to establish the efficacy, safety, and mechanisms of action of TACM interventions for bone fractures, ultimately informing evidence-based practices and healthcare policies in this domain.

## Conclusion

In conclusion, this systematic review highlights the potential benefits of TACM interventions for patients with bone fractures, including pain management, acceleration of bone union, and reduction of post-surgery complications. However, the quality of evidence for these interventions is currently limited, with the majority of studies demonstrating a high risk of bias. This emphasizes the need for more rigorous research designs and transparent reporting to improve the quality of evidence on TACM interventions for bone fractures.

The results of this systematic review demonstrated the varying efficacy and safety of different TACM modalities for patients with bone fractures. However, due to the heterogeneity of the included studies, it was not possible to conduct a meta-analysis. As a result, there is a lack of effect size and uncertainty regarding efficacy. Furthermore, the majority of the reviewed studies had a limited number of participants and a short follow-up duration. Therefore, it is recommended that future clinical studies on TACM treatments for patients with bone fractures address these issues in order to provide further insights.

The results of this review underscore the necessity for additional investigation to ascertain the effectiveness, safety, and underlying mechanisms of TACM interventions in the context of bone fractures. Subsequent studies should prioritize closing the existing gaps in evidence and policy to facilitate the secure and efficient integration of traditional and complementary practices into conventional healthcare systems. Furthermore, it is imperative for studies to disclose potential sources of bias, enabling an accurate assessment of the evidence quality.

## Acknowledgment

This study was financially supported by a grant from Shiraz University of Medical Sciences (No. 29382).

## Conflict of Interest

None.
